# Anti-*Acanthamoeba* Effects of Silver and Gold Nanoparticles and Contact Lenses Disinfection Solutions

**Published:** 2018

**Authors:** Maryam NIYYATI, Roghayeh SASANI, Mehdi MOHEBALI, Mahmoud GHAZIKHANSARI, Faranak KARGAR, Elham HAJIALILO, Mostafa REZAEIAN

**Affiliations:** 1. Dept. of Medical Parasitology and Mycology, School of Medicine, Shahid Beheshti University of Medical Sciences, Tehran, Iran; 2. Dept. of Medical Parasitology and Mycology, School of Public Health, Tehran University of Medical Sciences, Tehran, Iran; 3. Dept. of Pharmacology, School of Medicine, Tehran University of Medical Sciences, Tehran, Iran; 4. Rajaie Cardiovascular Medical and Research Center, Iran University of Medical Sciences, Tehran, Iran

**Keywords:** *Acanthamoeba*, *Acanthamoeba* keratitis, Nanoparticles

## Abstract

**Background:**

This study aimed to investigate the anti-*Acanthamoeba* effects of the most used marketed disinfecting solutions in Iran. Moreover, the efficacy of some nano-compounds was tested against pathogenic *Acanthamoeba*.

**Methods:**

The present study was conducted in the School of Public Health, Tehran University of Medical sciences, Tehran, Iran during 2015–2016. Cysts of *Acanthamoeba* T4 genotype (7 × 10^4^ /ml) mixed at the equal volume with contact lens solutions including Opti-free, Ginza, ReNu, Maxima, Light, and Cyclean for the recommended time by the manufacturers. Nano-silver and nano-gold compounds were also treated with the amoebae. Chlorhexidine 0.02% and normal saline were used as positive and negative controls, respectively. Dead and alive amoebae were determined using vital stain and suspension was cultured in non-nutrient agar. The entire process was repeated at least three times.

**Results:**

In none of the solutions in the manufacturer’s brochure recommended time, full cytotoxic effect was observed on the cysts of *Acanthamoeba*. Opti free express solution destroyed the cysts after 6 days. Nanosilver and nano-gold compounds showed no cytotoxic effect on the cysts of *Acanthamoeba*.

**Conclusion:**

None of the Nanoparticles compounds as well as contact lenses disinfecting solutions which studied was effective on *Acanthamoeba* cysts in the manufacturer’s brochure recommended time. However, continuing study on Nano-silver and Nano-gold compounds to find effective ingredients against *Acanthamoeba* are highly recommended.

## Introduction

*Acanthamoeba* spp. is free-living protozoa that exist in soil, fresh water, salt water, sea water, spa baths and Jacuzzis (1–5). *Acanthamoeba* spp. in its life cycle has trophozoite and cyst forms. During trophozoite encystation amoeba transforms to spherical structures and this is accompanied with synthesis of chemical and structural complex of cell wall which eventually with the maturity becomes double wall cysts (1). Cyst is caused protection against adverse environmental conditions and resistance to killing by freezing, sterilization and many antimicrobial agents (1–3).

*Acanthamoeba* spp. contain 20 genotypes that T4 genotype is the most common cause of corneal keratitis which is painful infection of the cornea and manifests often with tears, drooping eyelids, hyperemia, conjunctivitis, the destruction of the outermost layer of the cornea and photophobia. The symptoms lead to inflammation and redness, stromal infiltration, edema, blurred stroma, with severe eye pain, loss of epithelial and stromal abscess formation which eventually can lead to blindness (4, 6). In uncontrolled infections and severe cases, infection leads to enucleation.

*Acanthamoeba* keratitis (AK) treatment is very difficult and involves the use of drugs cocktail hourly included polyhexamethylene biguanid or chlorhexidine digluconate with hexamidine or isothionate propamidine which in addition to them chloramphenicol or neomycin should also be used to prevent bacterial infection (3). At the same time, the treatment takes months and there is the possibility of recurrence (3, 4).

Several factors are effective in Acanthamoeba keratitis infection. However, the most important factor is the use of contact lenses (1–5). Indeed, 95% of AK patients constitute contact lenses consumers (3). Wearers of contact lenses, depending on the cause divided into two cosmetic and therapeutic groups (7). Among the advantages of contact lenses are the mobility and natural movement in everyday life, doing different sports, and increasing wavelength range (8, 9). Nowadays due to increased number of consumers of contact lenses, these infections have become increasingly important in human health topic. Consumers of contact lenses mainly use two kinds of solutions: normal saline and commercial lens solutions which produced by different companies and lenses should be kept in it at least for 5 hours. Previous studies which studied common disinfectants in that period in the respective countries indicates that many of the solutions against *Acanthamoeba* are ineffective and some solutions are effective only against trophozoite form (10–12).

As there were no previous studies in Iran, the main aim of the present study was to investigate the anti-*Acanthamoeba* effects of the most used disinfecting solutions and nano compounds using counting and culture method.

## Materials and Methods

### Preparation of Acanthamoeba cysts belonging to T4 genotype

*Acanthamoeba* belonged to the genotype T4 obtained from a keratitis patient admitted in the Dep. of Parasitology and Mycology, Tehran University of Medical Sciences, Tehran, Iran in 2015. The sample was cultured in non-nutrient agar medium seeded with *Escherichia coli* and the plate was sealed with parafilm and stored at room temperature. The outgrowth of the amoebae was monitored by light microscopy in different days. After 14 d cysts were obtained. Amoebae were scrubbed from the surface of the plate using 5^cc^ distilled water, by pipette Pastori, and a suspension was prepared by number of 7 × 10^4^ amoeba/ml using hemocytometer.

### Amoebicidal effect of contact lens disinfecting solutions on Acanthamoeba cysts

From cyst suspensions at a density of 7×10^4^ and six studied solutions including Opti-free (Express), Ginza, ReNu (multiple), Maxima, Light, and Cyclean, in the same volume spilled in separate microtubes and microtubes lids were closed and shacked until well blended. Then any solution was stored at room temperature in recommended time by the manufacturers’ brochure (Table 1).

**Table 1:** Contact lens disinfection solution examined in the current study

***Solution***	***Ingredients***	***Time***	***Country***
Opti-free (Express)	Sodium chloride, Sorbitol, edetate disodium, Boric acid, Aminomethyl propanol, citrate and TETRONIC 1304 as cleaning agents, POLYQUAD (polidronium chloride) 0.001% and ALDOX (Myristamidopropyl dimethylamine) 0.0005% preservatives	6 h	Texas USA
GINZA	PHMB (0.0001%), HPMC, Poloxamer, Edentate disodium (0.05%), Sodium chloride, Boric acid and Borax	6 h	P. R. C
RENU – Multi Plus	HYDRANATE (hydroxyalkylphosphonate)boric acid, edetate disodium, poloxamine, sodium borate, sodium chloride preserved with DYMED (polyaminopropyl biguanide) 0.0001%	4 h	BAUSCH & LOMB (USA)
Maxima	PHMB	4 h	NETHER LANDS
Light	Polyhexanide 0.0001% w/v	4 h	ENGLAND
Cyclean	Polyhexanide 0.0001% w/v	4 h	ENGLAND

Chlorhexidine 0.02% used as a positive control and normal saline used as a negative control. After adding cysts suspension with concentrations of 7×10^4^ /ml with an equal volume of the studied solutions, in the recommended time by the manufacturers, vital stain (eosin 1%) added to them by equal volume to suspension and shacked until well blended.

Negative and positive controls were then evaluated to count the number of amoebae by hemocytometer. All cysts in the positive control were died and stained with eosin. After confirming reliability of controls, number of viable cysts was counted in each solution. This monitoring was continued for a week. Moreover, cysts of amoebae treated for a week with six contact lenses disinfecting solutions were cultured in non-nutrient agar and growth status was monitored during a month by microscopy. All assays were repeated three times.

### Efficacy of Nano compounds against Acanthamoeba

The compounds used in this study were obtained from faculty of Pharmacy, Tehran University of Medical Sciences and consisted of 13–10 nm nano-sized silver citrate, gold Nano-rads in 40 nm size with surfactant ctab and gold Nano-rads in 50 nm size with surfactant ctab. The protocol of treatment was the same as disinfecting solutions as mentioned above. The treatment time was set up as 0.5, 1, 2, 3, 4, 5, 6, 12, 24 h up to one week.

## Results

A pure culture of pathogenic *Acanthamoeba* cysts achieved in 10^th^ – 14^th^ day after culture. The dead cysts were detected by the pink to red color of cells and the alive cysts were detected by their colorless cytoplasm. Cysts treated with disinfection solutions including GINZA, ReNu, Maxima, Light, and Cyclean for the manufacturers recommended time, the vast majority of cysts were survived until seventh day (Table 2).

**Table 2:** Average number of live and dead *Acanthamoeba* cysts in seven consecutive days treating six contact lenses solutions by eosin staining

***Day***	***Cyclean***		***Light***		***Renu Multiplus***		***Maxima***		***Opt free Express***		***Ginza***		**Negative control		**Positive control	
	**Dead**	**Live**	**Dead**	**Live**	**Dead**	**Live**	**Dead**	**Live**	**Dead**	**Live**	**Dead**	**Live**	**Dead**	**Live (%)**	**Dead (%)**	**Live**
1	0.33	5.66	0	6.33	0	4	0	7.33	0	6.66	0	4.66	0	100	100	0
2	0	6.66	0	8	1	9	0	8.66	3.66	0.33	0	9	0	100	100	0
3	0	6.66	0	7.33	0	5.33	0	7	5	0.33	0	8.33	0	100	100	0
4	0	8.33	0.33	8.66	0	5.33	0	7	6.66	0	0	6.33	0	100	100	0
5	0	6.66	0	6.66	0	6.66	0	6	7.66	0	0	6.33	0	100	100	0
6	0	6	0	6	0.33	8.33	0	7.66	6.33	0	0	8	0	100	100	0
7	0.66	3.33	0	6	0	6.33	0.33	7.66	6.33	0	0.66	8	0	100	100	0

** Chlorhexidine 0.02% used as a positive control and normal saline used as a negative control (The table shows the treated days of six contact lenses solutions with *Acanthamoeba* T4 genotype)

**Fig. 1:**
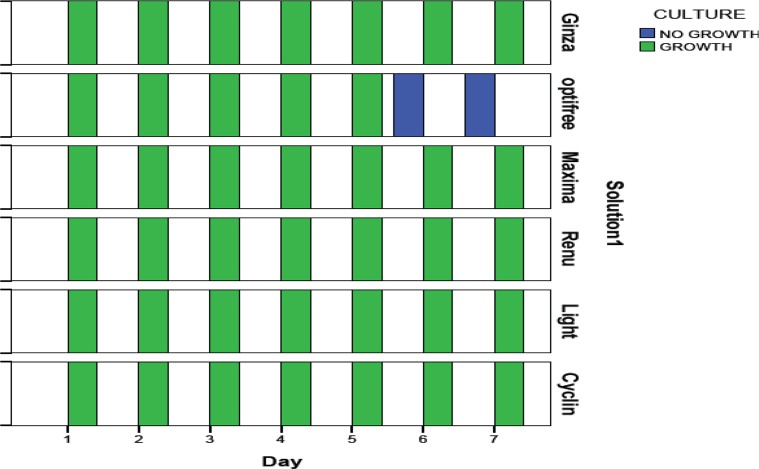
Results *of Acanthamoeba* cysts culture treated with six contact lenses disinfection solutions for 7 d (Growth in culture medium was observed in all solutions except Opti-free (Express) that on days 6 and 7 of treatment with cysts growth was not seen)

However, in OPTI-FREE (Express) solution after day 4 no alive cysts were observed by eosin staining and all observed cysts, stained with eosins which imply that they were dead. However, the growth in culture medium was observed in all solutions except Opti-free (Express). On days 6 and 7 of treatment, growth was not seen, which was differente with results of counting method of these two days (Fig. 1). Regarding nano-drugs in the current study was observed that until a week none had any cytotoxic effect on the cysts of *Acanthamoeba*.

## Discussion

*Acanthamoeba* spp. is the most common amoebae which found in the environment (3, 4). These amoebae in contrast to many physical and chemical factors such as heat, drought, freezing, disinfectants and UV show strong resistance. So far more than thousands of Acanthamoeba keratitis cases have been reported from around the world and given the increasing use of contact lenses; the figure will be higher (5). In Iran, among 142 patients with ocular keratitis referred to Tehran University of Medical Sciences, *Acanthamoeba* has been identified as the cause in of cases 34.5% (4). The main risk factor was the use of soft contact lenses along with the lack of hygiene in the lens storage (4). Using ophthalmic lenses has found considerable popularity among the people. But the major problem of people in the use of the contact lenses is not knowing the right way of maintenance and using lenses that will ultimately lead to eye problems. From points which individuals must follow, are replacement of lenses container every few weeks, removing the lenses at bedtime, caution when inserting and removing the lens in the eye and using lens solutions (9). The effectiveness of contact lens disinfection solutions in the prevention of AK is of utmost interest (11–13). Many studies evaluating the effectiveness of different types of contact lens disinfection have been done in America and Europe. However, very low number of such studies has been conducted in developing countries. Due to importance of this evaluation and the lack of such studies so far in Iran, this study was conducted in Iran on six common marketed contact lens disinfecting solutions.

In six investigated lens care solutions in our study, lethal property against *Acanthamoeba* was just mentioned in the manufacturer brochure of opti-free express. Two of the six solutions studied in this research, Opti free express and ReNu Multiplus, evaluated in previous studies, which reported similar results to ours (10, 12). The effectiveness of contact lens solutions such as Opti free express and ReNu Multiplus have evaluated against ophthalmic parasites including *Acanthamoeba castellanii* cysts and none of them killed *Acanthamoeba* cysts (10). Moreover, several contact lens care solutions including Ciba Vision AoSept Plus, Bausch & Lomb ReNu MultiPlus, Alcon Opti-Free, Ciba Vision Solo Care Aqua had no amoebicidal effects against *Acanthamoeba* Neff strain and two other keratitis strains (12). In our study also none of solutions in manufacturer’s recommended time in the brochure, (Opti-free express within 6 h and ReNu Multiplus within 4 h) had complete cytotoxic effect on *Acanthamoeba* belonging to T4 genotype cysts. In express opti-free solution, the complete lethal effect observed on cysts after 6 d, however, this is a long time and barely is possible for any of contact lenses consumers to put their lenses in the solution for this period. In the fourth day of treatment of parasite with Opti free solution, no alive cysts have been reported in counting method, but in culture, from the sixth day there was no growth, therefore the results of culture method are more reliable because the probability of errors in counting method is possible. Moreover, the OPTI-FREE® PureMoist® had limited effect on *Acanthamoeba* cysts of ATCC strain in the manufacturer recommendation time (14).

Six lens care solution was tested including All-in-One, All-in-One (Light), ReNu Multi-Plus, Optifree Express, Complete, and Solo-care soft and their results showed that only Allin-One showed effective against both trophozoites and cysts on the same time period (15).

To date targeting the cyst walls are the most effective strategies for increasing the efficacy of disinfecting solutions against *Acanthamoeba* (13). The use of chlorhexidine and cellulase enzyme could abolish viability of *Acanthamoeba* (13).

Our results showed that the ineffectiveness of contact lens solutions may be due to the lack of quality control in the production or import process or maintenance condition of in-store or exposure them to high temperatures.

Regarding nano-drugs lethal effect on cysts was not observed in 13–10 nm nano-sized silver citrate, gold Nano-rod with size 40 nm to with surfactant ctab and gold Nanorod with size 50 nm with surfactant ctab up to a week and the effect on *Acanthamoeba* cysts need to be further studied. Reviews of the synthesis of this material by an approach to investigation of effectiveness on *Acanthamoeba*, and needs to be investigated further.

## Conclusion

The contact lens care solutions tested in the present study were not effective against a clinical strain of *Acanthamoeba* T4 genotype. Thus, more studies to evaluate the efficacy of new enzymes and molecules against *Acanthamoeba* cysts are of high priority.
